# Irido-Choroiditis, Commonly Called Moon Blindness, in the Horse

**Published:** 1881-04

**Authors:** William O. Moore

**Affiliations:** Professor of Comparative Ophthalmology, Columbia Veterinary College


### ART. IX.—IRIDO-CHOROIDITIS, COMMONLY CALLED MOON BLINDNESS, IN THE HORSE.

VETERINARY science in ancient times received a dignity, not only from being practiced by the most celebrated of Latin poets, Virgil, but by being patronized by an emperor whose reign aided the cultivation of useful knowledge and the exertions of genius, considering them as the principal ornaments of his power. The horse being the most useful of all animals, and held in high regard by the people of olden time, it is no wonder that we find in their early writings descriptions of the various diseases to which it was subject.

The disease of which we are to speak seems to have attracted attention in very early times, and although not as old as the moon, dates nearly as far back. Moon-eye, or lunatic-eye, seems to have been the first name applied to this disease, from the prevailing opinion that the affection increased or decreased with the course of the moon, the eyes being muddy at the full moon, and at the new clearing up again. The blindness in these cases was not due to the direct lunar influence upon the eyes, but supposed to be due to some subtle influence on the system at large.

This notion of the old writers of the influence of the moon, is widespread, and not confined to horses, as we well know insane persons were called lunatic on the same ground.

Specific ophthalmia seems to have been the next name given, the belief having obtained that the disease was due to a specific virus acting in the system. Others called it constitutional ophthalmia, claiming that it was a disease of the general system, with a local manifestation in the eyes. Fearon, considering that gout was the primary cause, named it gouty ophthalmia. Sewell, of England, believing it arose from dentition, called it odontalgic ophthalmia; then, from its occurring at intervals, Percivall called it periodic ophthalmia. This last name has been used of late years more than the others. It is also a better name, yet is open to the objection that it conveys the impression of periodicity, which is not the fact. The disease is one continuous affair with exacerbations, slowly and surely progressing until the final cataract is produced.

This fact may be doubted, but can be proven by the use of the ophthalmoscope. When, to all appearances, the exterior of the eye is well, this instrument will show active inflammation of the interior, viz., choroiditis, etc. I would suggest as a name, irido-choroiditis, as the iris and choroid are the tissues principally involved.

*Etiology.*—Very many cases have been given by writers both of ancient and modern days, and still we are groping for the true light. All have, however, agreed that the moon has no influence in producing it, and that the name, "moon blindness," should be expunged from the nomenclature. There is no doubt that hereditary influence plays an important part in the causation, a fact noticed by the first writers. Taquetus says (1727), "This is an hereditary distemper, and therefore great care must be taken to *chuse* stallions that have good eyes." Marrimpoey,* considers this cause a very potent factor, and brings many facts to prove it, showing that stallions affected with blindness from this disease produce progeny almost invariably blind. This same fact has also been noticed by others, and in Ireland the prevalence of this disease is so accounted for. We have no doubt that in the majority of instances this is the primary cause, and all that is needed is an exciting cause to bring on the disease.

Rapid change of food has by very many been considered an exciting cause. It has been noticed in Lucerne, Switzerland, that

---

* "Receuil de Medical Veterinaire," 1829.

horses pastured on the mountains, where the provender is poor and scanty, the disease does not exist; but in the valleys, where the pasture is abundant and luxuriant, it is quite common even in horses not stalled The same conditions of plethora from overfeeding may give rise to it in horses constantly stalled. Gibson, one of the oldest writers, considers that in chewing oats "the muscles about the eyes are so strained that a defluction of rheum, or more blood than necessary is drawn towards the eyes by the motion aforesaid;" and suggests that the oats be stamped or ground. It has also frequently been found to occur after long-continued or repeated attacks of diabetes, which is often produced by feeding on mow-burnt or kiln-dried oats. This was noticed especially in 1816, when the British army was in France, when large numbers of horses were attacked with diabetes, with subsequent eye trouble. We have seen cases which seemed to have diabetes as an exciting cause. In the human subject we frequently find changes in the eye from this disease; cataract often, retinitis more rarely. The altered condition of the blood, due to the large loss of water, has much to do with this. It has been found to supervene on "grease," exhaustion from severe hæmorrhage, over-driving, hereditary influence as the primary, and any condition that depresses the system as the exciting cause.

*Pathology.*—Gibson, an old writer, says that moon blindness is due to "an obstinate stagnation in the small vessels of the tunica adnata, or white of the eye, and a relaxation of the small kernels that are seated at each of the angles of the eye; that the corrosiveness of the matter destroys the transparency of the cornea, so as to cause blindness." This, Bracken (1767) considers a very lame description of the distemper. This author is the first, so far as known, to call the disease by its proper name, and to appreciate the part of the eye involved, he named it *iritis.* Many years afterward, Pritchard again reminds the profession that the iris is the tissue primarily affected. Examinations of eyes, both in the acute and in the last stages of this disease, have been made. D'Arborval, in many cases, found, on making a section of the eye, absence of anterior chamber, adhesions of iris to lens, atrophy of iris fibres, in some instances bony deposits, the lens shrunken, and in most cases opaque; the vitreous changed to a citron color, and striated, being more fluid than normal. Hemmorrhages between choroid and selerotic are often found, except beneath that portion of the choroid called the tapetum-lucidum. An instance is recorded where the entire choroid was ossified, forming a bony cup. In some cases the optic nerve has been found atrophic. Other observers have noticed the same lesions, viz., those of irido-choroiditis. Excavation of the optic disc has never been observed.

*Symptoms.*—" Now they be called moon-eyes," says the old writer, Markham; "because, if the farrier do observe them, he shall perceive that at some times of the moon the horse will see very prettily, and at some times not at all. Now, the signs thereof are, when the horse's eyes are at the best they will look rather yellowish and dimme, and when at the worst they will look red, fiery and angry."

The symptoms differ with the stage of the disease and the severity of the attack; but taking a case in the beginning, one will usually get the following history:

Either the horse has had a slight intolerance of light for a day or two, or else none whatever, being apparently well stabled.

In the morning one eye will be found to be inflamed very much; the lids hot and swollen, great fear of light, increased secretion of tears, conjunctiva of lid reddened, nictitating membrane swollen and thrust forward to protect the eye from light, the eyeball retracted in orbit by posterior rectus.

On opening the lids the cornea will be found in some cases clear, in others hazy. When corneal trouble exists, it is of the interstitial variety; the iris, in most cases, loses its lustre; the pupil is sluggish or contracted; in many cases hypopion will be found in considerable quantity.

In severe cases the pupillary space is filled with lymph, the corpora nigra or soot-balls swollen, the whole eye having the appearance of violent iritis.

In milder cases the symptoms come on quickly. We find the same condition of the pupil, either sluggish or adherent, without any exudation in the anterior chamber. Ophthalmoscopic examination of the eye at such time will show large numbers of floating bodies in the vitreous, and in some instances patches of exudation in the choroid.

We have frequently seen the above appearances. The photophobia and acute inflammation may continue from one to four weeks, gradually subsiding ; the eye remaining, however, sensitive to light after all external evidences of disease have disappeared.

The horse is now considered by the owner well, and is used in the ordinary way.

In a period ranging from one to six months, the same train of acute symptoms reappear, usually in a more violent manner than at first ; this inflammation gradually subsides only to recur in the course of a few weeks, or perchance months. The outbreaks of active trouble come closer and closer together, until blindness closes the attack ; cataract being the final result.

We have watched cases during the stage of quiescence (when it was formerly supposed no disease existed), and with the ophthalmoscope have seen the choroiditis and vitreous changes in active progress, showing conclusively that it is continuous, and not periodic in the sense previously understood.

It is, in fact, an irido-chorciditis with exacerbations, resulting in cataract. After blindness occurs in one eye, the fellow eye usually becomes inflamed, following the same course as the first.

Some escape with only one blind eye, but the majority become diseased in both. We suggest as the cause of the disease in the fellow eye, sympathetic irritation through the ciliary nerves by reflex action, which fact now rests on a real and physiological basis, as recently confirmed by Mooren* and Rumpf.

The period during which the disease continues varies from three months to two years.

Many old writers were of the opinion that " wall-eyed " horses were exempt, but this has been found to be incorrect. Most all agree that the small sunken eye, technically called "pig-eye," is particularly liable ; this may be accounted for in part by the fact that these are usually animals of feeble constitution. This disease is rarely found under four years, commonly between four

* Centralblatt für de Med. Wissenschaft, Nr. 18, 1880.

and eight, rarely after, seeming to have a preference for the age of maturity. It is quite rare in Spain and Portugal, in considerable amount in England, France, and the United States, and very common in Ireland. Its frequency in the latter country is probably due to the impoverished condition of their animals and their poor stable facilities.

The *differential diagnosis* between this and catarrhal conjunctivitis at the onset, is necessary, and may be determined by the following points :

In conjunctivitis the patient usually has a general catarrh of the nasal mucous membrane; the symptoms come on gradually, and in *both* eyes; the secretion is mucus, the iris has its normal lustre, and the pupil acts. In the other disease the attack is sudden, in one eye only, the secretion only tears; the iris is sluggish or adherent, with some general febrile disturbance. The prognosis is very unfavorable.

*Treatment.*—The old works on farriery are full of methods of treatment, many of which are very novel and interesting.

Braken advises the following prescription :

Venice turpentine, three ounces.
Living millipedes, ½ gill.

The millipedes are to be bruised and mixed with the turpentine, of which a mass is to be made by mixing with flowers of brimstone ; out of this balls are to be made, and one given daily.

Ligature of the temporal artery has been advised, and by many old writers practiced. After a great array of prescriptions, Bracken has the frankness to confess that, had he a horse with this disease, he would not wait to try any of his methods, but first get rid of the animal.

This is, perhaps, the wisest thing to do. The treatment is very unsatisfactory, and it is doubtful whether very much can be done. We would suggest the following treatment as being the most rational :

When the eye is first attacked, the inflammation just beginning, the great object is to dilate the pupil so that no adhesions between the iris and lens will take place. This is accomplished best by sulphate of atropia, gr. 2 to 4, to an ounce of distilled water. This solution should be dropped into the eye four times a day.

The atropia relieves pain as well as having an action on the pupil.

To promote the absorption of the lymph and pus in the anterior chamber, and the infiltration of the cornea which often happens, the best thing is the application of warm water, bathing the eye one half-hour three times a day.

Where the hypopyon is very great and does not yield to the hot water, paracentesis of the cornea may be done, thus allowing the pus to escape, and at the same time diminishing the tension of the eye. Leeching may be resorted to in severe cases.

Febrile disturbance of the system must be treated in the usual manner.

A case is on record where accidentally a lancet was thrust through the cornea into the anterior chamber, the aqueous humor escaping; the eye immediately improved, and never became attacked subsequently.

We would advise the paracentesis to be tried; for the escape of exudation will certainly improve the eye, if it will not do permanent good.

Internally, we would advise the administration of iodide of potassium in moderate doses (gr. xl. t. i. d.), mixed with the food, together with the use of mercury, either by inunction or by the mouth; this latter drug to be continued till the gums are "touched."

The object of this internal treatment is to arrest, if possible, the choroiditis and other changes in the interior of the eye. When the inflammation has left the exterior of the eye, the atropine may be discontinued unless adhesion of the iris exist.

The internal medication should be continued as long as the floating bodies in the vitreous remain, stopping the medicine should there be an intolerance of the drug. We have watched several cases in which this plan of treatment was adopted, the ultimate result being very good.

This treatment will do much for this affection if adopted in the first attack, but promises less and less, the more chronic the disease.

In any case where the diseased eye is causing sympathetic irritation of the fellow, as shown by photophobia, redness, and inactivity of the pupil, it is advisable to perform the operation on the diseased eye called optico-ciliary neurectomy, which consists in thoroughly dividing the optic nerve, and especially the ciliary nerves which enter around it. This may be done and the eye-ball left in situ. This has been done on the human eye for this purpose with good effect. It will certainly do no harm, and may prevent the disease causing blindness in the fellow eye. Some old writers speak of persons, who, "put out one eye to save the other;" they, however, simply opened the eye and caused the evacuation of its contents, leaving the optic and ciliary nerves intact.

Notwithstanding our best endeavors, many cases will terminate in blindness.

WILLIAM O. MOORE, M.D.,
*Professor of Comparative Ophthalmology, Columbia Veterinary College.*

---

### BIBLIOGRAPHY.

MASCAL—London, 1545.

MARKHAM, GERVAISE—"Masterpiece," 1666.

GIBSON—"On Farriery," 1727.

BARTLET, J., Surgeon—"The Gentleman's Farriery," London, 1754.

WALLIS, THOMAS, Surgeon—"Dictionary of Farriery," Dublin, 1766.

BRACKEN, HENRY M.—"Treatise on the Art of Farriery," 1767, London. 2 vols.

LAWRENCE, RICH.—"Diseases of the Horse," London, 1801.

BLAIN, DELABERE—"Outlines of Veterinary Art, London, 1802. 2 vols.

MASON, RICHARD, M.D.—"Gentleman's New Pocket Farrier," Richmond, Va., 1825.

WHITE, JAMES—"Compend of Veterinary Art," London, 1822.

"THE VETERINARIAN,"—London, 1825 to 1840.

PERCIVALL, WILLIAM—"Hippopathology," London. 4 vols.

Since 1840, very little new material has been added to the literature of the subject.

